# Whole-genome sequencing and comparative analysis of heavy metals tolerant *Bacillus anthracis* FHq strain isolated from tannery effluents in Bangladesh

**DOI:** 10.3934/microbiol.2022018

**Published:** 2022-06-24

**Authors:** Farhana Haque, Ishrat Jabeen, Chaman Ara Keya, Sabbir R. Shuvo

**Affiliations:** Department of Biochemistry & Microbiology, School of Health & Life Sciences, North South University, Dhaka, Bangladesh

**Keywords:** Whole-genome sequencing, lead resistant, *Bacillus anthracis*, Bangladesh, *In-silico* analysis

## Abstract

Heavy metal contamination of the environment is a primary concern in Bangladesh. This study aims to characterize a novel heavy metal tolerant strain, *Bacillus anthracis* FHq, isolated from the tannery effluents of Savar, Bangladesh. The strain could tolerate up to 5 mM of lead nitrate, 2.5 mM of sodium arsenate, chromium chloride, cobalt chloride, 1.5 mM cadmium acetate, and 1 mM of sodium arsenite. Whole-genome sequencing analysis revealed that the genome of the strain is around 5.2 Mbp long, and the G + C content is 35.4%. Besides, FHq has genes c*adC, zntA, arsCR, czcD*, and c*hrA*, which confer lead, arsenic, cobalt, and chromium resistance, respectively. A total of nineteen other closely related and completely sequenced *B. anthracis* strains were selected based on average nucleotide identity along with the FHq strain for phylogenomic and pan-genome analysis. The phylogenomic analysis predicted the inter-genomic evolutionary relationship of the strain isolated from Bangladesh, and it was closely related to a strain isolated from China. Pan-genome analysis revealed that the FHq strain possesses 6045 pan genes, 3802 core genes, and 152 unique genes in its genomic content. Hence, the genetic information and comparative analysis of the FHq strain might facilitate identifying the mechanisms conferring high resistance to lead in *B. anthracis* strains isolated from Bangladesh.

## 1. Introduction

Industrial processes and various chemical compounds contain heavy metals and metalloids. The persistence of these metals and metalloids causes significant accumulation in the environment and negatively impacts animal health [1]. Human organs like the liver, lung, brain, and bones can be damaged from continuous exposure to heavy metals and metalloids. In Bangladesh, the dispersal of lead (Pb), cadmium (Cd), and chromium (Cr) in the environment are related to unplanned and rapid automation and urbanization [2]. Municipal waste, industrial effluents, pesticides, and chemical fertilizers cause heavy metals and metalloid pollution in the soil of industrial zones and rivers around Dhaka and Chattogram districts of Bangladesh. Therefore, a higher amount of lead, cadmium, chromium, and arsenic (As) were detected in these industrial zones and the tannery effluent in Bangladesh [3],[4].

*Bacillus anthracis* is a spore-bearing Gram-positive bacteria known for heavy-metal tolerance in different niches. Previously, several *Bacillus* species have been isolated from heavy metal contaminated sites [5]. The cell wall of Gram-positive bacteria serves as a natural barrier to heavy metals. Besides, teichoic and teichuronic acids present in the peptidoglycan are responsible for binding heavy metals [6]. Carboxyl and hydroxyl groups in macromolecules and bio-ligands like amide and sulfonamide are also involved in binding heavy metals in some *Bacillus* species [7]. The average genome size of *B. anthracis* is 5 to 6 megabase pairs [8]. The pan-genome of *B. anthracis* consists of roughly 3000 genes [9].

In this study, a strain of *B. anthracis* FHq was isolated from tannery effluents in Bangladesh. The objective of the study was to identify significant genes that are included in the resistance of heavy metals. In addition, the genomic database obtained from the study strain will provide insight into determining the mechanisms conferring high resistance to heavy metals of a *B. anthracis* strain isolated from Bangladesh. To the best of our knowledge, the whole genome sequencing data of a heavy metal tolerant *B. anthracis* strain FHq-isolated from Bangladesh is reported for the first time. Additionally, a comprehensive study of the metal resistance genes and a comparative analysis were conducted between the FHq strains and nineteen closely related and completely sequenced *B. anthracis* strains based on the average nucleotide identity (ANI) to identify unique genes for FHq.

## 2. Materials and methods

### 2.1. Bacterial strain

The bacterial strain was isolated from tannery effluent of Savar, Bangladesh, following the protocol described in [10] with few modifications. The sample was collected in a sterile glass bottle and transferred to the laboratory at North South University. The sample was serially diluted (10^−1^ to 10^−10^), and 100 µL of the sample was plated on the Lysogeny Broth (LB) plates (Himedia, India) supplemented with 1mM lead nitrate (Pb(NO_3_)_2_) (Merck, Germany) and grown for 24 hours at 37 °C. Individual colonies with distinct characteristics were collected and were grown in LB broth. For further studies, these bacterial colonies were stored at -80 °C in 50% (v/v) glycerol stock.

### 2.2. Heavy metal resistance and characterization of the novel strain

The strain was grown at 37 °C in the LB plate for 24–36 hours, supplemented with different concentrations (1 mM, 1.5 mM, 2 mM, 2.5 mM, 3 mM and 5 mM) of lead nitrate (Pb(NO_3_)_2_), sodium arsenate (Na_3_AsO_4_) (Merck, Germany), sodium arsenite (NaAsO_2_) (Merck, Germany), cobalt chloride (CoCl_2_) (Scharlab, Spain), cadmium acetate (Cd(CH_3_CO_2_)_2_.2H_2_0) (Qualikems,India) and chromium chloride (CrCl_3_) (Scharlab, Spain). Growth was observed after overnight incubation. LB plates without heavy metal supplementation were used as a control during each observation.

### 2.3. Whole-genome sequencing and annotation

The bacterial culture was grown overnight at 37 °C for DNA extraction. DNA extraction was performed using the Wizard Genomic purification kit (Promega, USA) according to the protocol provided by the manufacturer. The Illumina DNA Prep kit (Illumina, USA) was used to prepare the library by following the manufacturer's protocol. Illumina Miniseq technology (Illumina, USA) was used for sequencing the genome (Invent Technology Ltd., Dhaka, Bangladesh). Trimmomatic was used to remove the adapters from FASTQ sequences [11]. *de novo* assembly of the read to the contigs was done by using the Shovill (https://github.com/tseemann/shovill) (Galaxy Version 1.0.4 + galaxy1) pipeline, and Spades assembler was utilized to assemble the genome in the Galaxy server [12]. The annotation of the draft genome was performed by using the RAST server [13] and the National Center for Biotechnology Information (NCBI) Genome Annotation Pipeline (PGAP) [14]. The data for the contigs can be found in NCBI (PRJNA668995).

### 2.4. Identification of metal resistance, prophage sequences, and pathogenicity

The metal resistance genes were predicted from the RAST server data and compared with the genes in the BacMet database [15]. Phage Search Tool Enhanced Release (PHASTER) server was used to identify prophage sequences in the genome [16]. The PHASTER server predicts the prophage sequences according to the score on a scale of 150. Hit score > 90 is considered an intact or complete prophage sequence; < 70 is considered incomplete, and a score ranging between 70 to 90 is regarded as a questionable prophage sequence [16]. The PLSDB plasmid database was used to check the presence of plasmid [17]. The pathogenicity of the strain was checked using PathogenFinder 1.1 [18]. In every case, the draft genome sequence of *B. anthracis* FHq was input in FASTA format, and the default parameters of the servers were used.

### 2.5. Phylogenomic tree construction

A total of nineteen *B. anthracis* strains (S1 Table) were collected from the NCBI database based on their average nucleotide identity (ANI) (96–100%) calculated in the EDGAR software (16) (S2 Table). Later, Type Strain Genome Server (TYGS) [19] was used with the default parameter to construct the phylogenomic tree along with the *B. anthracis* FHq strain. TYGC used Genome BLAST Distance Phylogeny (GBDP) strategy to contract the phylogenomic tree.

### 2.6. Comparative genomic analysis

Each of the nineteen *Bacillus* strains was analyzed using the RAST server to find the heavy metal resistance genes and compared to the heavy metal resistance genes of the *B. anthracis* FHq strain. Later, pan-genome analysis was performed by using Bacterial Pan-Genome Analysis (BPGA) software [20]. The BPGA software also predicted the Clustered Orthologous Groups (COG) of proteins by comparing the protein sequences of the genomes.

### 2.7. Function of the predicted hypothetical protein involved in metal tolerance

The unique gene sequences identified in the pan-genome analysis of FHq were further analyzed using pBLAST. The functions of the hypothetical proteins, encoded by some unique genes, were predicted using Pfam [21], HHpred [22], and Conserved domain database-CDD [23]. The Pfam server implements the Hidden Markov Model (HMM) algorithm to predict the domains. HMM is a statistical model used to describe the evolution of observable events that depend on internal factors and are not directly observable. The HHpred server builds an alignment of homologs for the query sequence by multiple iterations of PSI-BLAST searches against the non-redundant database from NCBI. The CDD uses RPS-BLAST, a variant of PSI-BLAST, to scan a set of pre-calculated position-specific scoring matrices with the given protein query.

## 3. Results

### 3.1. General Characteristics of B. anthracis FHq

**Figure 1.**
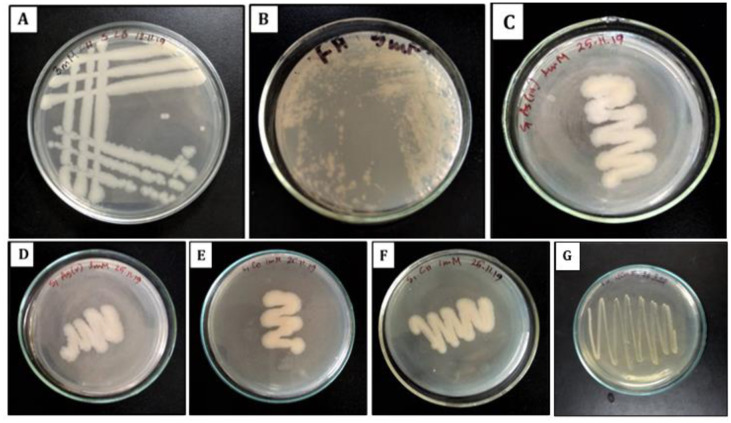
Growth of the bacterium at different concentrations of lead nitrate (Pb(NO_3_)_2_). *Note: (A) 3 mM; (B) 5 mM concentration of the salt. Growth of the bacterium at (C) 1 mM sodium arsenate (Na3AsO4); (D) 2.5 mM sodium arsenite (NaAsO2); (E) 2.5 mM cobalt chloride (CoCl2); (F) 2.5 mM chromium chloride (CrCl3); (G) 1.5 mM cadmium acetate (Cd(CH_3_CO_2_)_2_). The growth was observed by using triplicates for each concentration.

The isolated strain was found to tolerate up to 1 mM of sodium arsenite, 1.5 mM cadmium acetate, 2.5 mM of sodium arsenate, 2.5 mM cobalt chloride, 2.5 mM chromium chloride, and 5 mM concentration of lead nitrate (Figure 1).

The genome length of FHq was calculated to be 5238597 bp, the G + C content was 35.4%, and the N50 value of the assembly was 572044 (S3 Table). The RAST server annotated the genes and divided them into 332 subsystems, where the majority of the subsystems are categorized as protein metabolism, amino acid and derivatives, and carbohydrate (S1 Figure). The RAST server also predicted that the genome contains 112 rRNAs and 5544 coding sequences (CDS) (S3 Table). No plasmid was detected by using The PLSDB database. However, the PHASTER server could predict three genome regions that contain prophage sequences (S4 Table). The first region of the sequences was similar to Phage Lister 2389, a native bacteriophage of *Listeria* species [24]. The second and third regions of the sequences were matched with Phage *Bacill* vb BhaS 171, and the Phage *Bacill* Waukesha92, respectively. Later, results from PathogenFinder 1.1, showed that the probability of FHq being a human pathogen is 0.782, where 1 is the highest value of probability.

**Table 1.** Predicted genes for lead and other heavy-metal resistance in *B. anthracis* FHq.

Heavy metals	Genes	Functions of the proteins
Lead	*cadC*	Cadmium efflux system accessory protein Function: Regulates the *CadA* gene involved in Pb, Cd, and Bi resistance.
	*zntA*	Lead, cadmium, zinc, and mercury transporting ATPase; Copper-translocating P-type ATPase Function: Activates to response stress signal in bacteria
Arsenic	*arsR*	Arsenical resistance operon repressor
	*arsC*	Arsenate reductase thioredoxin-coupled, Low molecular weight phosphatases (LMWP) family
	*acr3*	Arsenical-resistance protein ACR3
Cobalt	*czcD*	Cobalt/zinc/cadmium resistance protein CzcD
Chromium	*chrA*	Chromate transport protein

### 3.2. Determination of the putative heavy metal resistance genes from the FHq genome and phylogenomic analysis

Protein BLAST (pBLAST) was performed on the genomic data from the RAST server to identify the functions of the proteins. Later, the BLAST result was compared to the BacMet database- a database for antimicrobial, biocide, and metal resistance genes. The *zntA* and *cadC* genes involved in lead resistance; and *acr3, arsC, arsR* genes involved in arsenic resistance were detected in the genome of FHq. Furthermore, *czcD* and *chrA* involved in cobalt and chromium resistance, respectively, were also detected in the genome of FHq (Table 1) and (Tables S5 and S7).

The CadC regulates the *cadA* gene; *cadA* gene is responsible for lead, cadmium, and bismuth resistance [25],[26]. ZntA protein has the highest selectivity for Pb^2+^, Zn^2+,^ and Cd^2+^ [27],[28]. The *acr3* gene encodes the arsenical resistance protein that works as an arsenical resistance membrane transporter [29] Besides, the *acr3* gene is a part of the ars operon that contains *arsC* and *arsR* genes. The *arsR* gene codes ArsR protein, which is a trans-acting regulatory protein. Moreover, ArsC protein reduces arsenate [As(V)] to arsenite [As(III)] [29]. *czcD* is a transporter gene that influences Zn^2+^ and Cd^2+^ resistance and is required to activate the *czc* operon [30]. The *chrA* gene encodes chromate transporting protein ChrA, responsible for the inducibility of the resistance to chromium compounds [31].

**Figure 2.**
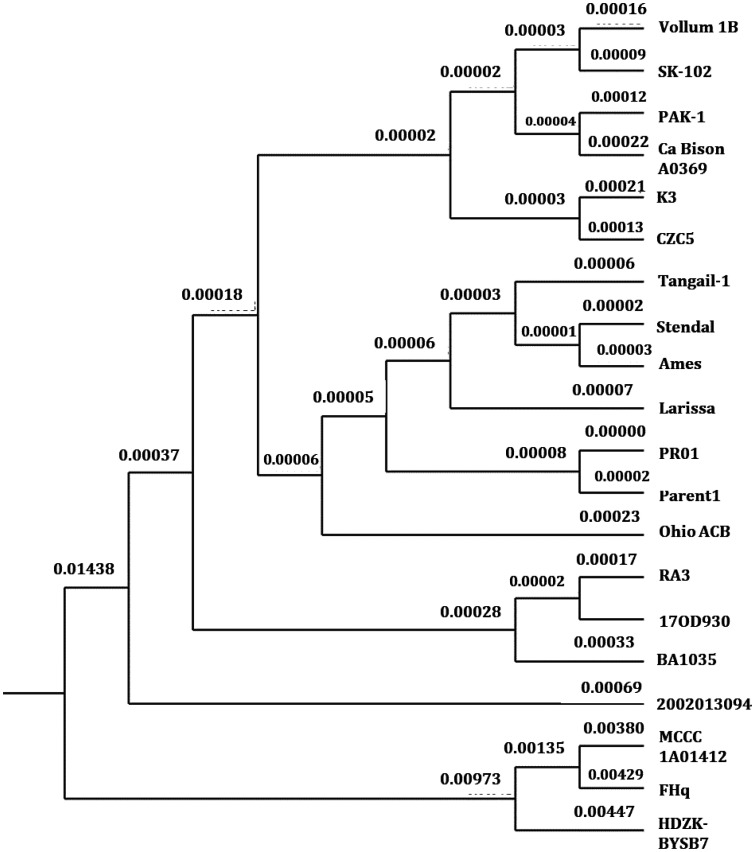
Phylogenomic tree of the twenty *B. anthracis* strains based on the whole genome sequences. *Note: The strains were analyzed and constructed by TYGS by using the GBDP method. The branch length was measured in terms of the GBDP formula of d5. The branch lengths were taken up to 5 decimal points.

The phylogenomic tree was constructed using the type strain genome server by inputting the complete genome sequences of the *B. anthracis* strains (S1 Table). The FHq strain belongs to a completely different branch along with the MCCC 1A01412 (Figure 2).

### 3.3. Pan-genome analysis of the twenty B. anthracis strains

Pan-genomes of twenty different strains of *B. anthracis* were analyzed using Bacterial Pan-genome analysis (BPGA) software. Genes present in all strains of the data set are termed core genes. Genes present in some strains are the accessory genes, and the genes specific or unique for a single strain are annotated unique genes. Core, accessory, and species-specific gene families represent the pan genes in that analysis, also known as the pangenome [32]. Genes present in the other members of the dataset but absent in a particular one are exclusively absent genes. The total gene families of the compared strains range from 4744 to 6045 (S8 Table). All the strains share 3802 core gene families in the dataset. The FHq strain has the maximum number of gene families (6045) and the highest number of unique gene families (152) among all the analyzed strains in our study. In contrast, the lowest number of gene families present in the *B. anthracis* Ames strain was 4744. Additionally, accessory genes varied for each strain, ranging between 892 to 1116.

Later, the COG analysis of the pan-genome showed that most of the proteins in the genome, especially the core genes, are involved in metabolic activities from the pan-genome analysis (Figure 4). Most of the unique genes are involved in information processing and storage categories.

**Figure 3.**
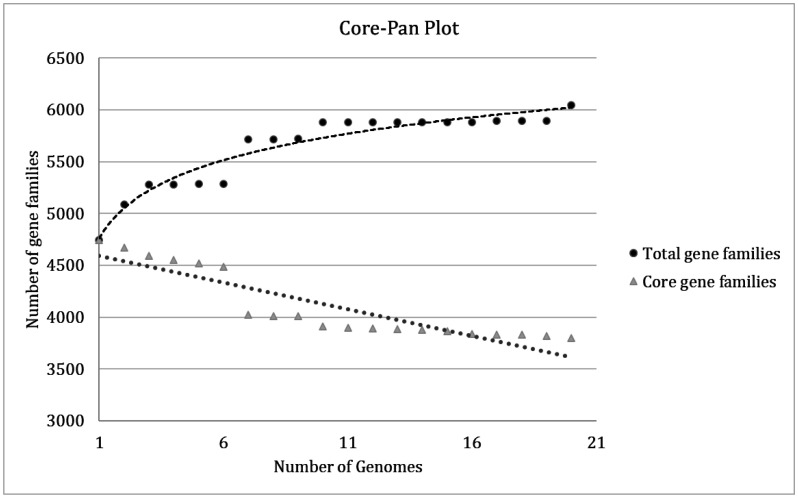
A pan and core genome plot of *B*. *anthracis*.

The plot indicates how the number of total gene families rises and declines in the core gene families with each consecutive inclusion of a *Bacillus anthracis* genome. The black regression line indicates pan-genome, and the grey regression line indicates core-genome.

### 3.4. Function of the predicted hypothetical proteins

Among the 152 unique gene sequences predicted in pan-genome analysis in the FHq genome, 76 (50%) genes were predicted to encode hypothetical proteins. Among the used servers, only HHpred predicted that the hypothetical protein (HP12) has structural and sequence homology with the *cnrR* gene-a nickel-cobalt resistant gene [33]. This *cnrR* gene has metal sensing and signals transduction activities towards Ni (II) and Co (II). Besides, the functions of 12 other hypothetical proteins were predicted (S6 Table). Those hypothetical proteins are involved in DNA binding, transcription regulation, and virulence.

**Figure 4.**
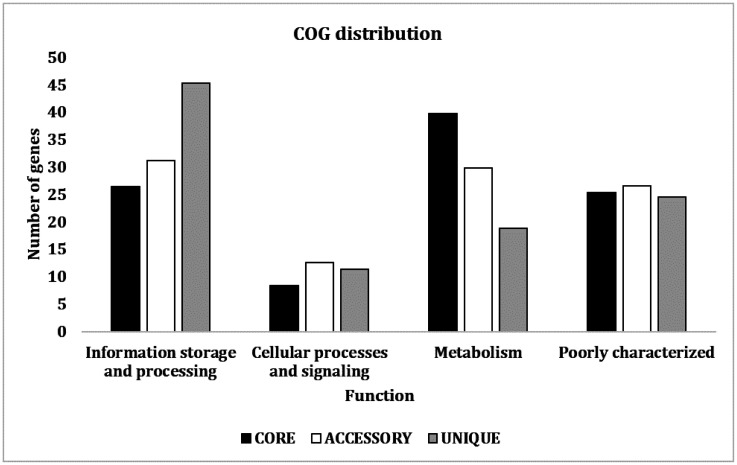
Distribution of COG from the pan-genome analysis of the selected *B. anthracis* strains. *Note: The X-axis represents the respective functions of the genes, and the Y-axis represents the number of genes.

## 4. Discussion

Heavy metals and metalloid contamination are increasing worldwide, including in Bangladesh [2]. The adverse effects of heavy metals used in the industries have been known for a long time. In addition, the typical tannery wastewaters contain Pb, Cu, Fe, Na, Cr, Cd, Zn, and Ni-like heavy metals [3],[4]. Heavy metals are mixed with the nearby areas' soil and water through the tannery discharges, causing health hazards to humans, animals, plants, and aquatic lives.

This study was conducted on genotypic and phenotypic characteristics of a novel *B. anthracis* FHq strain isolated from tannery effluent of the Savar area, Bangladesh. The lead tolerance level of the FHq strain was double that of arsenic, chromium, and cobalt. The MIC of lead nitrate was 5 mM in the *B. anthracis* FHq strain. In contrast, previous studies reported that the average lead tolerance level of *Bacillus* species is 3 mM lead [34]–[36].

**Figure 5.**
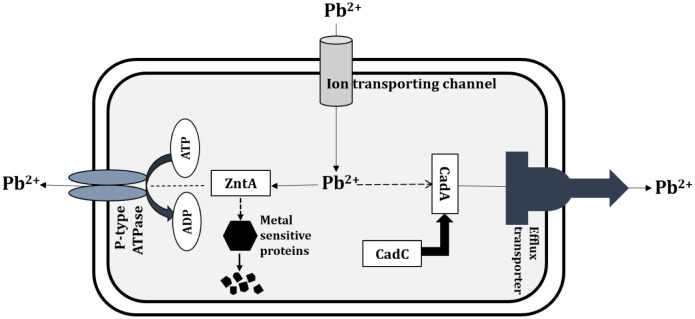
Proposed lead (Pb) resistance pathways in *B. anthracis* FHq strain.

Whole-genome sequencing data of the strain FHq was found consistent with the phenotypic data obtained from our study. The genomic analysis could predict a few heavy metal tolerance genes, as described in the result section. Among all the heavy metal resistant genes, the *cadC* and *zntA* genes have been found in the genome of the FHq strain that is likely to be responsible for lead resistance (Table 1). In addition, the *cadC* gene encoding cadmium efflux system accessory protein regulates the *cadA* gene that is responsible for conferring resistance to lead, cadmium and bismuth during *in vitro* analysis [26]. The *zntA* gene codes for lead, cadmium, zinc, and mercury transporting ATPase along with copper-translocating P-type ATPase. Therefore, based on the above-mentioned discussion, a lead-resistance pathway for the FHq strain might be proposed, as shown in Figure 5.

The genome of FHq also contains *arsC, arsR*, and *acr3* genes for arsenic; *czcD* for cobalt; and *chrA* gene for chromium resistance. The *arsR* gene encodes a trans-acting transcriptional repressor protein that binds to the promoter region of *ars* operons. The interaction of this protein with arsenite dissociates the repressor protein from the DNA and allows transcription of the operon [29]. Arsenate reductase thioredoxin-coupled enzyme from the low molecular weight proteins (LMWP) family is encoded by the *arsC* gene (Table 1). In *B. subtilis* species, the *arsC* gene catalyzes the intracellular reduction of arsenate to arsenite. Arsenite is then extruded from cells through an efficient and specific transport system that provides arsenic resistance [29]. Besides, chromium resistance in bacteria is primarily performed by a specific efflux system that pumps chromate out of the cell, thereby lowering the intracellular concentration [37].

The unique hypothetical proteins of the FHq strain were further analyzed. The *In-silico* study by the HHpred server predicted only one metal resistance gene (Denoted as HP12, shown in (S6 Table) from the hypothetical proteins. This gene has sequence similarities with the *cnrR*, also known as *cnrX*, a gene associated with metal binding, metal sensing, signal-transducing, and specifically nickel-cobalt resistance. The *cnrR* gene encodes CnrR protein that is found in the cytoplasm. Structurally, the CnrR protein contains six histidine residues, whose spatial arrangement in the primary amino acid sequence is identically conserved in the NccX protein. The NccX protein is a cation efflux protein involved in nickel-cobalt-cadmium resistance [38].

Among the studied strains, *B. anthracis* MCCC 1A01412 is the nearest neighbor of the FHq strain in the phylogenomic tree. The strain was isolated from sediment of the South China sea (S1 Table). Both FHq and MCCC 1A01412 have similar heavy metal resistant genes (S5 Table). Furthermore, a comparative study was performed on all the strains selected for phylogenomic analysis. Except for *chrA* (absent in the 17OD930 strain), all the other metal resistance genes identified in FHq are present in the genomes of the other nineteen strains of the dataset (S5 Table).

In the pan-genome analysis, the strain FHq carries the highest number of unique gene sequences of 152 to be exact, of which 76 genes (50%) encode hypothetical proteins. A total of 30 gene sequences out of the total 76 genes (~40%) are unique for *B. anthracis* species, and 46 (60%) are commonly found in multiple species of *Bacillus*. There are 15 phage sequences encoding phage head, tail, and portal proteins and a phage replisome organizer N-terminal domain-containing proteins commonly found in multiple *Bacillus* species. The rest of the unique sequences encode several enzymes, regulatory proteins, and toxins. As per the COG database, the highest number of unique genes found in the FHq strain are involved in metabolism (Figure 4). However, a significant portion of the gene codes for proteins with unknown functions.

## 5. Conclusion

Novel *B. anthracis* strain FHq revealed the presence of multiple metal resistance genes. Those metal resistance genes might be involved in the metal resistance pathways to increase the metal resistance capacity of the FHq strain compared to other *B. anthracis* strains. Besides, *In-silico* analysis of the hypothetical proteins predicts the function of a metal resistance protein. Lastly, the phylogenomic analysis imparts the evolutionary relationship of FHq with other *B. anthracis* strains isolated around the world.
