# Supplementary material for: Whole-genome sequencing and comparative analysis of heavy metals tolerant Bacillus anthracis FHq strain isolated from tannery effluents in Bangladesh
Source: AIMS Microbiol. 2022 Jun 24;8(2):226–38. doi: 10.3934/microbiol.2022018 (PMC9329874; doi:10.3934/microbiol.2022018)
Supplement: Supplementary file 1 [file microbiol-08-02-018-s001.pdf]

AIMS Microbiology

AIMS Microbiology, 8 (2): 226–238.
DOI: 10.3934/microbiol.2022018
Received: 22 January 2022
Revised: 18 May 2022
Accepted: 27 May 2022
Published: 22 June 2022

http://www.aimspress.com/journal/microbiology

Research article

Whole-genome sequencing and comparative analysis of heavy metals tolerant *Bacillus anthracis* FHq strain isolated from tannery effluents in Bangladesh

Farhana Haque, Ishrat Jabeen, Chaman Ara Keya and Sabbir R. Shuvo *

Department of Biochemistry & Microbiology, School of Health & Life Sciences, North South University, Dhaka, Bangladesh

* **Correspondence:** Email: sabbir.shuvo@northsouth.edu; Phone: 88-0255668200.

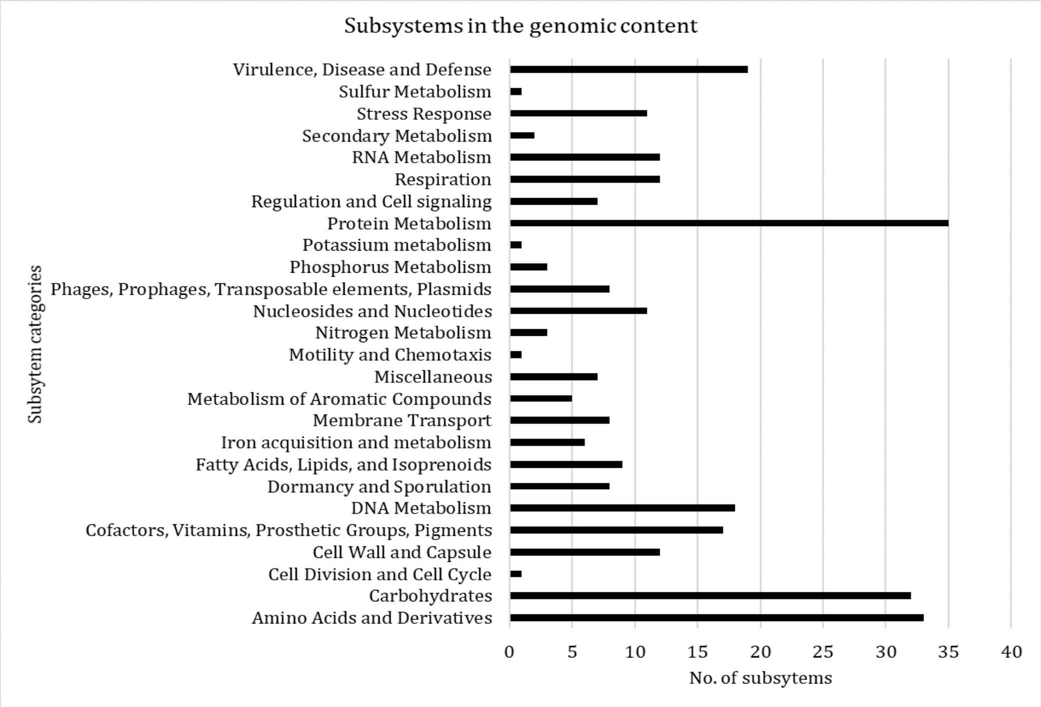

S1 Figure. Subsystems in the genomic content generated by the RAST server.

S1 Table. List of the *B. anthracis* strains that have been used for phylogenetic analysis and comparative analysis.

Sl.	Accession no.	Organism	Origin	Source	Base pairs	G+C (%)*	No. of proteins*
1	NC_003997.3	*Bacillus anthracis* Ames	Texas	Cow	5227293	35.38	5474
2	CP029323.1	*Bacillus anthracis* 17OD930	Switzerland	*Bos taurus*-placenta	5213297	35.03	2926
3	NZ_CP009700.1	*Bacillus anthracis* BA1035	South Africa	Human	5210596	35.35	5453
4	NZ_CP010322.1	*Bacillus anthracis* Ca Bison A0369	Canada	Bison	5229224	35.38	5477
5	NZ_CP009331.1	*Bacillus anthracis* K3	South Africa	Human	5228730	35.38	5473
6	NZ_CP009341.1	*Bacillus anthracis* Ohio ACB	USA: Ohio	Pig	5222171	35.38	5461
7	NZ_CP009325.1	*Bacillus anthracis* PAK-1	Pakistan	Sheep	5221627	35.37	5453
8	NZ_CP009697.1	*Bacillus anthracis* RA3	France	Bovine	5213209	35.36	5465
9	NZ_CP009464.1	*Bacillus anthracis* SK-102	USA	Wool from Pakistan	5230088	35.38	5467
10	NZ_CP009328.1	*Bacillus anthracis* Vollum 1B	USA	*Homo sapiens*	5229969	35.38	5472
11	NZ_CP009902.1	*Bacillus anthracis* 2002013094	USA	Soil	5321900	35.39	5523
12	NZ_AP018443.1	*Bacillus anthracis* CZC5	Zambia	*Hippopotamus amphibius*	5219278	35.37	5455
13	NZ_CP012730.1	*Bacillus anthracis* Parent1	USA	No data	5228660	35.38	5471
14	CP012720.1	*Bacillus anthracis* PR01	USA	No data	5228656	35.38	5471
15	CP014179.1	*Bacillus anthracis* Stendal	Germany	*Bos bovis*	5226939	35.38	5471
16	CP026608.1	*Bacillus anthracis* HDZK-BYSB7	USA	Bark of cypress	5294779	35.43	5289
17	NZ_CP012519.1	*Bacillus anthracis* Larissa	Greece	Ovis: blood	5226878	35.38	5471
18	CP031643.1	*Bacillus anthracis* MCCC 1A01412	China: South China Sea	Sediment	5291783	35.41	5369
19	CP015779.1	*Bacillus anthracis* Tangail-1	Tangail, Bangladesh	Soil	5227292	35.38	5473
20	PRJNA668995	*Bacillus anthracis* FHq	Dhaka: Bangladesh	Soil	5238928	35.2	5394

*Note: G+C (%) and no. of proteins were taken from the TYGS result.

S2 Table. ANI matrix from EDGAR.

	FHq	MCCC1A 01412	HDZK-BYSB7	20020 13094	CZC5	BA1 035	RA3	17OD 930	Ca Bison A0369	Vollum 1B	SK-102	K3	Stendal	Larissa	Parent 1	PR01	Tangail 1	Ames	Ohio ACB	PAK1
**CZC5**	96.83	96.88	97	99.81	100	100	100	99.9	99.95	99.96	99.97	100	99.96	99.96	99.96	100	99.96	100	100	99.97
**Larissa**	96.84	96.87	97	99.81	99.9	100	100	99.9	99.95	99.95	99.97	100	99.99	100	99.98	100	99.99	100	100	99.97
**Ames**	96.84	96.87	97	99.81	99.9	100	100	99.9	99.95	99.95	99.96	100	100	99.99	99.99	100	99.99	100	100	99.97
**Tangail-1**	96.84	96.87	97	99.81	99.9	100	100	99.9	99.95	99.95	99.96	100	99.99	99.99	99.99	100	100	100	100	99.97
**PR01**	96.84	96.87	97	99.81	99.9	100	100	99.9	99.96	99.96	99.96	100	99.99	99.99	99.99	100	100	100	100	99.97
**Parent1**	96.84	96.87	97	99.81	99.9	100	100	99.9	99.96	99.96	99.97	100	99.98	99.98	100	100	99.98	100	100	99.97
**Stendal**	96.84	96.86	96.99	99.81	99.9	100	100	99.9	99.94	99.95	99.95	100	100	99.98	99.98	100	99.98	100	100	99.96
**SK-102**	96.83	96.88	97.01	99.82	99.9	100	100	99.9	99.96	99.98	100	100	99.97	99.97	99.97	100	99.97	100	100	99.97
**Vollum 1B**	96.84	96.88	97	99.81	99.9	100	100	99.9	99.96	100	99.99	100	99.97	99.96	99.96	100	99.97	100	100	99.97
**Ohio ACB**	96.84	96.87	97	99.8	99.9	100	100	99.9	99.95	99.96	99.96	100	99.97	99.96	99.97	100	99.97	100	100	99.96
**PAK-1**	96.85	96.88	97.01	99.81	99.9	100	100	99.9	99.96	99.96	99.97	100	99.96	99.96	99.96	100	99.96	100	100	100
**Ca Bison A0369**	96.84	96.87	97	99.81	99.9	100	100	99.9	100	99.96	99.96	100	99.96	99.96	99.96	100	99.96	100	100	99.98
**K3**	96.84	96.88	97.01	99.81	99.9	100	100	99.9	99.95	99.96	99.96	100	99.96	99.96	99.96	100	99.96	100	100	99.96
**17OD930**	96.84	96.87	96.99	99.8	99.9	100	100	100	99.92	99.93	99.93	99.9	99.93	99.93	99.93	99.9	99.93	99.9	99.9	99.93
**RA3**	96.85	96.87	96.98	99.79	99.9	100	100	100	99.91	99.92	99.93	99.9	99.93	99.93	99.93	99.9	99.93	99.9	99.9	99.93
**BA1035**	96.83	96.86	96,98	99.78	99.8	100	100	100	99.9	99.9	99.9	99.9	99.9	99.91	99.9	99.9	99.9	99.9	99.9	99.91
**20020 13094**	96.89	96.88	97.01	100	99.8	100	100	99.9	99.89	99.89	99.89	99.9	99.9	99.89	99.9	99.9	99.9	99.9	99.9	99.9
**HDZK-BYSB7**	98.92	96.87	100	96.98	97	97	97	97	97.01	97.02	97.03	97	97.01	97.04	97.05	97.1	97.06	97.1	97.1	97.08
**MCCC 1A01412**	99.19	100	98.99	96.83	96.9	97	97	97	96.88	96.91	96.92	97	96.91	96.92	96.95	97	96.93	97	97	96.97
**FHq**	100	96.87	96.88	98.88	96.8	97	97	96.9	96.84	96.89	96.89	96.9	96.88	96.89	96.89	96.9	96.9	96.9	96.9	96.92

S3 Table. General features of *Bacillus anthracis* FHq.

Intital Reads	
R1	
Total read	754907
Sequence length	151
R2	
Total read	754907
Sequence length	151
Statistic	General features
Sequence size (bp)	5238928
Number of contigs	56
GC content (%)	35.2
Shortest contig size	201
Median sequence size	1404
Mean sequence size	93552.3
Longest contig size	661180
N50 value	572044
L50 value	5
No. of subsystems	332
rRNAs	112
Coding sequences	5544

S4 Table. List of phage sequences.

Sl.	Region length (kb)	Score	Most common phage*	Accession number	GC %
1	33.2	90	Phage Lister 2389	NC003291	34.36
2	22.1	110	Phage Bacill vb BhaS 171	NC030904	36.30
3	31.8	70	Phage Bacill Waukesha92	NC025424	35.73

* Note: Common phage: The phage with the highest number of proteins is most similar to those in the region.

S5 Table. Presence of heavy metals in twenty strains of *B. anthracis.*

	Lead (Pb)		Arsenic (As)			Cobalt	Chromium
	cadC	*zntA*	*arsR*	*arsC*	*acr3*	*czcD*	*chrA*
FHq	+	+	+	+	+	+	+
MCCC 1A01412	+	+	+	+	+	+	+
HDZK-BYSB7	+	+	+	+	+	+	+
Ames	+	+	+	+	+	+	+
Tangail-1	+	+	+	+	+	+	+
PR01	+	+	+	+	+	+	+
Parent1	+	+	+	+	+	+	+
Stendal	+	+	+	+	+	+	+

Continued on next page

	Lead (Pb)		Arsenic (As)			Cobalt	Chromium
	cadC	*zntA*	*arsR*	*arsC*	*acr3*	*czcD*	*chrA*
SK-102	+	+	+	+	+	+	+
Vollum 1B	+	+	+	+	+	+	+
Ohio ACB	+	+	+	+	+	+	+
PAK-1	+	+	+	+	+	+	+
Ca Bison A0369	+	+	+	+	+	+	+
K3	+	+	+	+	+	+	+
17OD930	+	+	+	+	+	+	-
RA3	+	+	+	+	+	+	+
BA1035	+	+	+	+	+	+	+
2002013094	+	+	+	+	+	+	+
CZC5	+	+	+	+	+	+	+
Larissa	+	+	+	+	+	+	+

* Note: (+)= Present, (-)= Absent.

S6 Table. Predicted unique hypothetical proteins of *B. anthracis* FHq strain with an E-value <0.1.

Sl#	Protein	Predicted protein	Molecular functions	Subcellular location
1	HP1	DnaB-like Helicase	Unwinding DNA during replication Hydrolase activity Ion binding	Cytoplasmic Protein
	MKRIEDNIWHAFGEPLKFGFGTYLTAKAKVDLNPIRSIFEVLGILAKYPQLLLNPRMTLCEDDFVQTF HQMVFSAIKDIVCKDNSIKTLTYVDIDNHLSKDKELYELWLYCNGIDYMRDAIELSNKHTFKIHYRE LKKHTLMQHFACAGVDISSVHDYLQTDIPLLSRSEKRVRTLTYEELESELIPLGKGNDDMQQLLSDQ QQLIDTHISILMKRKQELQSLQDKLK			
2	HP6	Pneumococcal surface protein A	Virulent activities	Extracellular Protein
	MNKKLLTTLTCFALFMGLAACSSNETASTSKESKAEQEAQKEAEKTKKAEEKGTVKREKAEQETAE KQRKQQEESTKKEQPTTTSGNTIKGLKDELPIGMPFKDYLAKKKSLNVENPFSISLGKGNIGSVLQAQ DGVLVVCIDGEKIFDLKTFKNVDEAKEYEKSLKIKQ			
3	HP10	ROK	DNA binding protein.	Cytoplasmic Protein
	MNNHSENAAQKIREIAMEFMRKNEKKQFQRKDLEAYIDNQFEATEGSKTGALNRLLLRNEENGIFQ VGRGIYLYDPTAEKEEYQFLEGIQKIMDDAFERVRNSLKNIEVVDYLTEEDLEAIIKTNEVLKMKEKI DAILKVKS			
4	HP12	Metal-binding protein	Nickel and cobalt resistance, metal binding, metal sensor, signal transduction	Cytoplasmic Protein
	MLRSFIIILAFLVLSFSSTVFVEAKSNMTSYEDKNTFHYKHNYKQLYKFLNISKEEYSKSIQNGKSLGE IAAEQGVHEKELIVFLKKEVLKQLHQQWKFGKLNTRTYKGIKGHVDDFVKDDIYFKYKEFKRNTSG			

Continued on next page

Sl#	Protein	Predicted protein	Molecular functions	Subcellular location
5	HP15	Prophage LambdaLm01, Antigen B	No data	Cytoplasmic Protein
	MNDFINILHSELKQIHKESYYEIAKTTADMPYLVYTVNDDKEPWGRKNIMLTIDIYGTSAHLSKIDELITKLESNLHRKRLCSAEFGAAISYISSQKVPDSDPNIIRKEVRFILRTYFKQ			
6	HP21	Transcription regulator Reg576	Transcription regulation, plasmid establishment DNA binding, gene repression in Gram-positive bacteria	Extracellular Protein
	MDKQYLVTVAPIQSNSATKKKNPLSASDRKNIKVSPETLNKIKAICTMKDRKINELIHEMLDYYITNKLNSNEQNNLLLRR			
7	HP26	DNA Topoisomerase 3-Alpha	Double Holliday-Junction Dissolution	Cytoplasmic Protein
	MYKCPICKRHTLKDYIDMDTQEIYYMCDNKDCPDYGKQMTDIEIEE			
8	HP27	NuDix hydrolase	Catalyzes the hydrolysis of nucleoside diphosphates linked to other moieties, X, requires divalent cation for signal transduction	Cytoplasmic Protein
	MFIVNVEGAIRKNDKWLVIERSKKEEHAGGWLSLVGGKLDIEGNFSDILERTVK			
9	HP32	Bestrophin domain protein	Calcium-activated chloride channel. Macular degeneration.	Inner-membrane Protein
	MVHYNNQNSLHQIFTLKGTVIRDIFPQILLYICVSTVITVIHYYYATIRMNQTPWVIVGGSLGLLLVFRTNTAYDRYWEGRKLFGTIGACSRNLAVSFLCYWDSEGKKTDQEQLKFLHLLIAFPKIAKGHLRDEKDLSEIKELLNVCSEKEKEMLEQSMHLPISIVLMLKTILSKGLKTGQIHPNAIINMETDLNNLLTAVGGCDRIKTTPIPFAYFAHIKILLLIFCGTLPIGLVDSLGWVTVLATMFISFAFIGIEAIGVEIEDPFGQDPNDLPLEGICIGVETNLLNLYNQNGLLEVMDMNQVEKSCNH			
10	HP33	Hypothetical protein	Tetrapeptide repeats	Outer Membrane Protein
	MKKFLMSFSILLIATLLFGCSNETFDKAEEQGKLALANKEYTNANASFEIALKEKKDEEVQTLQKQASKMEKALIAKEKLDIDNAIKLFDQVSTMKDGLETVKNEAKKEKETLTQYKDKRENYNKELQTAQDLLSEKKFDESKVILTKVQQETTSDKMFTEQNQKVTEQLEKLEKEKNAAVEEEKRQEEQKKAAEQKKKQQAKEQQKQTAQSSKQKLSPTDAENKVRKTLNITNNSKVYCEYEGVEGEEYLIHVYQIVSDEEGGHTATYGWFNVNSNTGEVRKM			
11	HP38	Antimicrobial peptide LCI	No data	Outer Membrane Protein
	MFKKLVVGALAAGIALTGGIGAASASTEGVDNTNQVKPIKTLIGDYYNYDRGFSYHDVAGRPDIYRTYDVYSSNGIFDNSFYSEGRTWYFKGVASTNWDGSAYGHYEARK			
12	HP53	Lin0334 protein	No data	Periplasmic Protein
	MRKLSKKAQMKQDLLQQLEIGGLQGMHYVDLVDDYMTMFDVKNKLAREMKKNGPMIEWQNSDSQKGVKANPATKEFRETNKRMTELLKVLGLKEPVYEGNDDDDDI			
13	HP58	Transcription regulator Reg576	Transcription regulation, plasmid establishment DNA binding, gene repression in Gram-positive bacteria	Periplasmic Protein
	MTYFYTYLGRTPITDNSDLNTTTEKTMHAPEKKQFKNQQGSIKIPKQSKEELEALMKITNTKFTHEIIDLLINHYVNNKLTPEQQSKFKLITDI			

S7 Table. Predicted genes and respective sequences for lead, arsenic, chromium, and cobalt resistance in *Bacillus anthracis* FHq.

Heavy metal	Genes	Protein sequence
Lead (Pb)	*cadC*	MAGNKVETPQETCSQTIIHEEVVDQVKQTIPTDESLSKVAELFKVLGDRTRTRILHALFE<br>AEMCVCDLAYLLGMTQSSISHQLRVLKQAKLVKNRKEGKVVYYSLADQHVIHIFEQA<br>FEHVNEEE
	zntA	MNEQKEANLQISGMTCAACANRIEKGLKKVEGVHDANVNFALEKTKIMYDPQKTNPQ<br>QFKEKVESLGYGIVSDKAEFTVSGMTCAACANRVEKRLNKLEGVNGATVNFALESAT<br>VDFNPDEINVNEMKSAITKLGYKLEVKSDEQDESTDHRLQEIERQKKKFIISFILSFPLLW<br>AMVSHFSFTSFIYLPDMLMNPWVQLALATPVQFIIGGQFYVGAYKALRNKSANMDVL<br>VALGTSAAYFYSVYLSIQSIGSSEHMTDLYFETSAVLITLIILGKLFEAKAKGRSSEAIKK<br>LMGLQAKTATVVRDGTEMKILIEEVVAGDIVYVKPGEKIPVDGEIVEGKSAIDESMLTG<br>ESIPVDKTIGDVVIGSTMNKNGFLKVKATKVGRDTALAQIIKVVEEAQGSKAPIQRVAD<br>QISGIFVPVVVVIAIITFAVWMIFVTPGDFGGALEKMIAVLVIACPCALGLATPTSIMAGS<br>GRSAEYGILFKGGEHLEATHRLDTVILDKTGTVTNGKPVLTDVIVADGFNEEEILRLVG<br>AAEKNSEHPLAEAIVEGIKEKKIDIPSSETFEAIPGFGIESVVEGKQLLIGTRRLMKKFDINI<br>EEVSKSMEELEREGKTAMLIAINKEYAGIVAVADTVKDTSKAAITRLKKMGLDVVMIT<br>GDNTQTAQAIAGQVGIEHVIAEVLPEGKAEEVKKLQAQGKKVAMVGDGINDAPALAT<br>ADIGMAIGTGTDVAMEAADITLIRGDLNSIADAIFMSKMTIRNIKQNLFWALAYNGLGI<br>PIAAFGFLAPWVAGAAMAFSSVSVVLNALRLQRVKLK
		MNSEVKTLQVQKSISHPSLWDTLKKHYELIFAIASGIFILAGWLFTKNDVMNVGITCYIL<br>AYIVGGYAKAKEGIEDTIEEKELNVEMLMLFAAIGAAMIGYWAEGAILIFIFALSGAME<br>SYTLSKSQKEISALLDLQPEEALRISNGTEERVPVGRLQINDIILIKPGERVPADGTIHSGE<br>TNIDEAAITGEPIPNEKKHGDEVFAGTVNLRGAIEVKITKPSDQTLFQKIIRLVQSAQSEK<br>SPSQLFIEKFEGTYVKGVLLVVALMMFVPHFLLDWSWNETFYRAMILLVVASPCALVA<br>AITPATLSAISNGARNGILFKGGIHLERLASVKAIAFDKTGTLTEGKPTVTDVYVRENMT<br>EKEVLSITAAIESHSTHPLAESIVKYAQYAYDITLKKPENVEDVTGFGLKGIFESKAYKIG<br>KADFIGEETKTFHNGVSASLEKEGKTVVYISDEDGILGLIALKDTLRQETIAAIRELQNIG<br>VEAIMITGDNEETAKAIAAESNIKEYYASCLPETKVATIKKLKEKYGTVAMVGDGINDA<br>PALATASIGVAMGEGTDVALETADVVLMKNELSRLSQAIRLSKRMNRIVKQNVIFSLA<br>VIAMLICSNFLQFLALPFGVIGHEGSTILVILNGLRLLKGNN
Arsenic (As)	*arsR*	MTQDFQLYEKKFKALADQKRLEIMYELCQRGQTCVCDLTEIFEMTQSKLSYHLKILLD<br>AGLIVKETKGTWSYYDLNDTEVNNLLSEELCCIFRKTGKGSCC
	arsC	MENKKTIYFLCTGNSCRSQMAEAWGKQYLGDKWNVYSAGIEAHGVNPNAIKAMNEV<br>NIDITNQTSDIIDANILNRADLVVTLCSHADSVCPSTPPDVNRVHWGFDDPAGKEWSEF<br>QRVRDEIGERIKRFSETGE
		MNEVNIDITNQTSDIIDANILNRADLVVTLCSHADAVCPSTPPHVNRVHWGFDDPAGKE<br>WPEFQRVRDEIGERIKRFSETGE
	acr3	MKRLSFLDRYLTLWIFLAMAVGIGLGFVFPSVVDGLNTLQVGTTSIPLAVGLIVMMYPP<br>LAKVRYEEMGRVFKDVKVLVLSLVQNWIIGPVLMFVLAIIFLPDKPEYMVGLIMIGLAR<br>CIAMVIVWNDLADGDKEYAAGLVAFNSVFQMLFFSIYAYVFVTVIPKWLGIEGAIVDIT<br>MIEVAKSVFIYLGIPFIAGMLTRFVLVKLKGRQWYEKVFIPKISPLTLIALLFTIIVMFSLK<br>GEMIVSVPLDVVRIAIPLLIYFIVMFFVSFFMGRKIGANYPVTTTLAFTAGSNNFELAIAV<br>AVGVFGIHSGAAFAAVIGPLVEVPVMIALVNVAFWFKRKYFNDQPV

Continued on next page

Heavy metal	Genes	Protein sequence
Cobalt (Co)	*czcD*	MGHSHDHGHSKNKKALLLAFLLTTSFMIAEVIGGFVTNSLALLSDAGHMLSDAVSLAL<br>SLLAFKLGEKTATTAKTYGYKRVEMLAALCNGVVLIIISIYIFIEAIRRFKEPVEIASNGM<br>LIIAVLGLLINILSAWILMRGGDVKGNLNLRSAFLHVLGDLLGSVGAIIAALLIKFFGWT<br>AADAIASILVSILVIISGWRVTRDTVHILMEGAPQNINVEEVKSTLLNITIVKEVHDLHV<br>WSVTSDFQVLTCHLIIKGNETQSVLKEATEVLKEKFHVEHVTIQVEIDGEFHHSETTCKV
Chromium (Cr)	*chrA*	MKNNKYTFHTLLEIFLVSFKLGLTSFGGPVAHLGYFHHEYVQKRKWMDERSYGDLVA<br>LSQFLPGPASSQVGMGVGLLRGGLLGAIISWIGFTLPSVLVLVFFASFLNQLDLGSAGWI<br>HGLKLVAVAIVAHAIWGMAQKLTPDRNRATIAIATAAIALLWPSSWTQVILIIISGFIGW<br>FLYRNQPISQSQNIKVPISKKIAVSCLVLFFGLLLLLPILRPFSYYIALFDSFYRSGALVFGG<br>GHVVLPLLEGEFVQNGMMTKEQFLAGYGLTQAMPGPLFTFASYIGAVLNGTLGAILAT<br>IAIFLPAFLLVIGVLPFWDSVRKISFIQGALLGVNAAVVGILLAAFYDPIWTSTIMNAVDF<br>VFASLLFCLLAFWKTPPWVIVILGAFGGYILSIL

AIMS Press

© 2022 the Author(s), licensee AIMS Press. This is an open access article distributed under the terms of the Creative Commons Attribution License (http://creativecommons.org/licenses/by/4.0)
